# Decreased Expression of miR-21, miR-26a, miR-29a, and miR-142-3p in CD4^+^ T Cells and Peripheral Blood from Tuberculosis Patients

**DOI:** 10.1371/journal.pone.0061609

**Published:** 2013-04-16

**Authors:** Katja Kleinsteuber, Kerrin Heesch, Stefanie Schattling, Malte Kohns, Claudia Sander-Jülch, Gerhard Walzl, Anneke Hesseling, Ertan Mayatepek, Bernhard Fleischer, Florian M. Marx, Marc Jacobsen

**Affiliations:** 1 Department of Immunology, Bernhard-Nocht-Institute for Tropical Medicine, Hamburg, Germany; 2 Department of General Pediatrics, Neonatology, Pediatric Cardiology, University Children’s Hospital, Duesseldorf, Germany; 3 Department of Paediatrics and Child Health, Desmond Tutu Tb Centre, Faculty of Health Sciences, Stellenbosch University, Cape Town, South Africa; 4 Division of Molecular Biology and Human Genetics, MRC Centre for Molecular and Cellular Biology, DST and NRF Centre of Excellence for Biomedical TB Research, Faculty of Health Sciences, Stellenbosch University, Cape Town, South Africa; 5 Department for Pediatric Pneumology and Immunology, Charité - Universitätsmedizin, Berlin, Germany; The Ohio State University, United States of America

## Abstract

The vast majority of *Mycobacterium tuberculosis (M. tuberculosis)* infected individuals are protected from developing tuberculosis and T cells are centrally involved in this process. MicroRNAs (miRNA) regulate T-cell functions and are biomarker candidates of disease susceptibility and treatment efficacy in *M. tuberculosis* infection. We determined the expression profile of 29 selected miRNAs in CD4^+^ T cells from tuberculosis patients and contacts with latent *M. tuberculosis* infection (LTBI). These analyses showed lower expression of miR-21, miR-26a, miR-29a, and miR-142-3p in CD4^+^ T cells from tuberculosis patients. Whole blood miRNA candidate analyses verified decreased expression of miR-26a, miR-29a, and miR-142-3p in children with tuberculosis as compared to healthy children with LTBI. Despite marked variances between individual donor samples, trends of increased miRNA candidate expression during treatment and recovery were observed. Functional *in vitro* analysis identified increased miR-21 and decreased miR-26a expression after re-stimulation of T cells. *In vitro* polarized Interleukin-17 positive T-cell clones showed activation-dependent miR-29a up-regulation. In order to characterize the role of miR-29a (a described suppressor of Interferon-γ in tuberculosis), we analyzed *M. tuberculosis* specific Interferon-γ expressing T cells in children with tuberculosis and healthy contacts but detected no correlation between miR-29a and Interferon-γ expression. Suppression of miR-29a in primary human T cells by antagomirs indicated no effect on Interferon-γ expression after *in vitro* activation. Finally, classification of miRNA targets revealed only a moderate overlap between the candidates. This may reflect differential roles of miR-21, miR-26a, miR-29a, and miR-142-3p in T-cell immunity against *M. tuberculosis* infection and disease.

## Introduction

Approximately one third of humankind is infected with *Mycobacterium tuberculosis* (*M. tuberculosis*), the causative agent of human tuberculosis. Specific cellular immunity, especially CD4^+^ T cells, is capable of containing the pathogen in the vast majority of *M. tuberculosis*-infected individuals. These latently *M. tuberculosis*-infected individuals (LTBI) do not develop clinical symptoms of tuberculosis after infection, but remain at increased risk of developing active disease especially during the first years after *M. tuberculosis* infection. The exact mechanisms of how CD4^+^ T cells prevent the development of active disease remain elusive.

Animal models and human genetic studies point towards a crucial role of IFNγ expressing T helper type (T_H_) 1 cells in protective host immunity against tuberculosis [1]. Recent studies showed that ‘polyfunctional’ T cells characterized by the expression of multiple cytokine (i.e. IFNγ, TNFα, IL-2) were protective [2], [3] whereas single-cytokine (i.e. either TNFα ορ ΙΦΝγ) expressing T_H_1 cells were pathognomonic [4], [5]. Other T-cell subpopulations, like Interleukin-17 producing T_H_17 cells, may also influence disease susceptibility in tuberculosis, but previous studies revealed contrary results [6]–[11] Thus the quantity of a single T-cell cytokine, e.g. IFNγ, is not sufficient as a marker for protection against tuberculosis.

Little is known about the role of MicroRNAs (miRNAs) in containing *M. tuberculosis* infection and preventing disease progression. MiRNAs have been shown to fine-regulate target genes in eukaryotic cells, including immune cells, by modulating protein expression at the posttranscriptional level [12]. The maturation of miRNAs into functional regulators comprises three main steps: I) processing of the primary-miRNA transcripts (pri-miRNA) into the precursor miRNA (pre-miRNA) by the nuclear RNase III enzymes Drosha and DGRC8, II) export into the cytosol and subsequent processing into 22 bp duplexes by the cytosolic RNase III enzyme DICER and III) loading of the mature miRNA into the RNA-induced silencing complex (RISC), where it binds to the 3′-untranslated region (3′-UTR) of the target mRNA. MiRNAs exert their functions mainly by degradation of the target mRNA or inhibition of translation (reviewed in [13]). They play important roles in many physiological processes including the development and regulation of the immune system (reviewed in [14]). In T cells, miRNAs have already been shown to regulate several processes e.g. cytokine expression, polarization of distinct T-cell lineages, homeostasis of T cells, and T-cell receptor signaling [15]–[20]. Indications of immune pathological effects of miRNA-mediated regulation come from malignancies and autoimmune diseases (reviewed in [14]), but the role of miRNAs in chronic infectious diseases like tuberculosis is hardly defined. Recent studies identified miRNA (miR)-29 as a central non-redundant suppressor of IFNγ [21] and increased miR-29 expression promoted susceptibility against mycobacterial infections [22].

The aim of this study was to identify and characterize miRNAs involved in immunity against tuberculosis. For this purpose we initially compared expression of 29 pre-selected immune-related miRNAs in CD4^+^ T cells of tuberculosis patients, healthy LTBI, and non-*M. tuberculosis* infected individuals (PPDneg). A follow-up study was then performed to determine the expression of candidate miRNAs in whole blood of children with tuberculosis and healthy contacts.

## Results

### Identification of Differentially Expressed miRNAs in CD4^+^ T Cells of Tuberculosis Patients and LTBI

**Figure 1:**
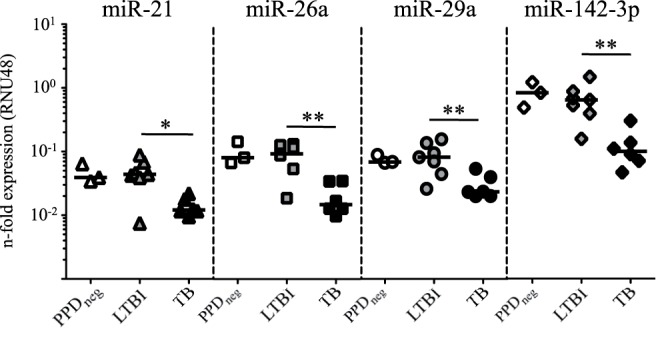
Four miRNAs are differentially expressed in CD4^+^ T cells from tuberculosis patients and LTBI. Expression of miR-21 (triangles), miR-26a (squares), miR-29a (circles), and miR-142-3p (diamonds) in CD4^+^ T cells from tuberculosis patients (TB) (black symbols, n = 6), LTBI (grey symbols, n = 7), and PPD negative healthy controls (PPDneg) (open symbols, n = 3). Median expression of candidate miRNAs relative to the housekeeping gene RNU48 is shown. Significant differences are indicated as asterisks (* for p<0.05; ** for p<0.01, Mann-Whitney U-test).

We compared the expression of 29 miRNAs in CD4^+^ T cells from peripheral blood of tuberculosis patients, LTBI, and non-infected control donors (PPDneg). MiRNAs were selected according to expression of T cells [23], [24]. Analyses were restricted to CD4^+^ T cells to avoid confounding influences of cellular heterogeneity on RNA expression [25]. For these analyses acute tuberculosis patients on diagnosis (i. e. prior to onset of chemotherapy) were included. 17 of 29 miRNAs were detectable according to defined criteria [for details see material and methods]. Median expression levels and standard deviations of all determined miRNAs are shown in Table 1. Four miRNAs, namely miR-21, miR-26a, miR-29a, and miR-142-3p, were differentially expressed between tuberculosis patients and LTBI (P = 0.035, P = 0.005, P = 0.008, and P = 0.002, respectively), whereas no differences were detected between LTBI and PPDneg (Table 1 and Figure 1A). Differentially expressed miRNAs showed decreased expression in CD4^+^ T cells from tuberculosis patients as compared to LTBI and PPDneg. Notably the level of differentially expressed miRNAs correlated markedly in T cells from individual donors (miR-21/miR-26a, P = 0.001; miR-21/miR-29a, P = 0.002; miR-21/miR-142-3p P = 0.001; miR-26a/miR-29a P<0.0001; miR-26a/miR-142-3p P<0.0001; miR-29a/miR-142-3p P<0.0001) (data not shown). This rendered a common cause of decreased miR-21, miR-26a, miR-29a, and miR-142-3p of CD4^+^ T cells from tuberculosis patients likely.

**Table 1:** Median Expression of miRNAs in PPD_neg_ donors, LTBI and tuberculosis patients.

	PPDneg		LTBI		Tuberculosis	
	Median	SD	Median	SD	Median	SD
let-7a	0,0008	0,0006	nd	nd	nd	Nd
miR-15a	0,0005	0,0003	0,0003	0,0013	0,0003	0,0008
miR-17-3p	0,1401	0,0311	0,1259	0,0403	0,071	0,02
miR-21	0,0393	0,0161	0,0438	0,0252	0,0121	0,0047
miR-23a	0,00005	0,00005	nd	nd	nd	Nd
miR-25	0,0032	0,0005	0,0027	0,0083	0,0015	0,0027
miR-26a	0,0808	0,0409	0,0928	0,0411	0,0148	0,0113
miR-27a	0,0016	0,001	0,0025	0,0054	nd	Nd
miR-29a	0,0687	0,0122	0,0815	0,0467	0,0233	0,0137
miR-30a*	0,728	0,0443	0,07	0,0294	0,036	0,0982
miR-98	nd	nd	nd	nd	nd	Nd
miR-99b	0,0003	0,0001	nd	nd	nd	Nd
miR-101	0,0008	0,0004	0,0014	0,001	nd	Nd
miR-106a	0,1478	0,0215	0,1185	0,0455	0,08	0,0222
miR-106b	0,0047	0,0011	0,003	0,0016	0,0018	0,0028
miR-122	nd	nd	nd	nd	nd	Nd
miR-126	0,0022	0,0007	0,0011	0,0013	0,0023	0,0055
miR-142-3p	0,8261	0,3628	0,6404	0,417	0,1	0,0909
miR-143	nd	nd	nd	nd	nd	Nd
miR-144*	nd	nd	nd	nd	nd	Nd
miR-146a	0,3294	0,0417	0,3099	0,1966	0,2069	0,1075
miR-150	2,459	0,7761	3,8087	1,6389	2,0281	1,3939
miR-155*	nd	nd	nd	nd	nd	Nd
miR-181a-1	0,001	0,0003	0,0007	0,0009	nd	Nd
miR-191	0,1224	0,366	0,0691	0,022	0,0558	0,0526
miR-203	nd	nd	nd	nd	nd	Nd
miR-218	nd	nd	nd	nd	nd	Nd
miR-223	0,8505	0,7369	0,1425	0,3888	0,1249	0,8014
miR-455-5p	nd	nd	nd	nd	nd	Nd
RNU48^1^			na	na	na	Na

1 control; nd: miRNA not detectable in at least two donors.

### Differential Expression of Candidate miRNAs in Blood of Children with Tuberculosis, LTBI, and PPDneg

**Figure 2:**
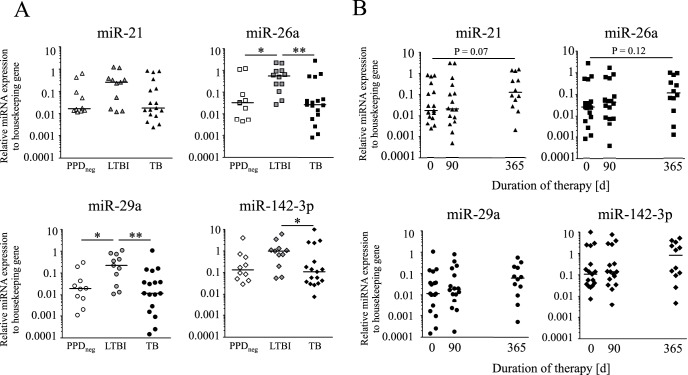
MiRNA candidate expression of peripheral blood cells from children with tuberculosis and LTBI. Expression of miR-21 (triangles), miR-26a (squares), miR-29a (circles), and miR-142-3p (diamonds) in whole blood is shown for children with tuberculosis (TB, black symbols), healthy latently *M. tuberculosis* children (LTBI, grey symbols), and PPD negative contacts (PPD_neg_, open symbols) (**A**) as well as for children with tuberculosis under therapy and recovery (**B**). Each symbol indicates miRNA candidate expression for an individual donor relative to ‘housekeeping’ control RNU48. Significant differences are indicated as asterisks (* for P<0.05; ** for P<0.01, Mann-Whitney U-test). Exact P-values (Mann-Whitney U-test) are indicated for tendencies.

Limited donors numbers included for CD4^+^ T-cell miRNA expression analyses prompted us to perform a follow-up case/control study in children with tuberculosis, LTBI, and PPDneg contacts. Here isolation of CD4^+^ T cells was not feasible because of the limited sample volume. Therefore miRNA candidate expression in whole blood was determined. Although the expression of miRNAs was heterogeneous between individuals, we detected markedly lower expression of miR-26a (P<0.01), miR-29a (P<0.01), and miR-142-3p (P<0.05) in children with tuberculosis as compared to children with LTBI (Figure 2A). For miR-21 the same trend was identified without reaching significant levels. Notably, miR-21, miR-26a, miR-29a, and miR-142-3p expression levels were comparable between children with tuberculosis and PPDneg children. MiR-26a and miR-29a were lower (both P<0.05) in PPDneg as compared to children with LTBI (Figure 2A). Time course analyses of CD4^+^ T cells from selected tuberculosis patients revealed markedly increased miRNA candidate expression during the first months of chemotherapy (data not shown). Therefore we determined miRNA expression in children with tuberculosis also during treatment (3 months after onset of therapy) and after recovery (12 months after onset of therapy) (Figure 2B). We identified trends of increased miRNA candidates expression (P = 0.07 for miR-21; P = 0.12 for miR-26a) after recovery as compared to active disease (Figure 2B). We concluded that miRNA candidate expression in peripheral blood of children largely reflected differences between CD4^+^ T cells. Next we analyzed a possible role of miRNA candidates during T-cell activation and differentiation.

### Expression of miR-21, miR-26a, miR-29a and miR-142-3p after in vitro Re-stimulation of Naïve T Cells

**Figure 3:**
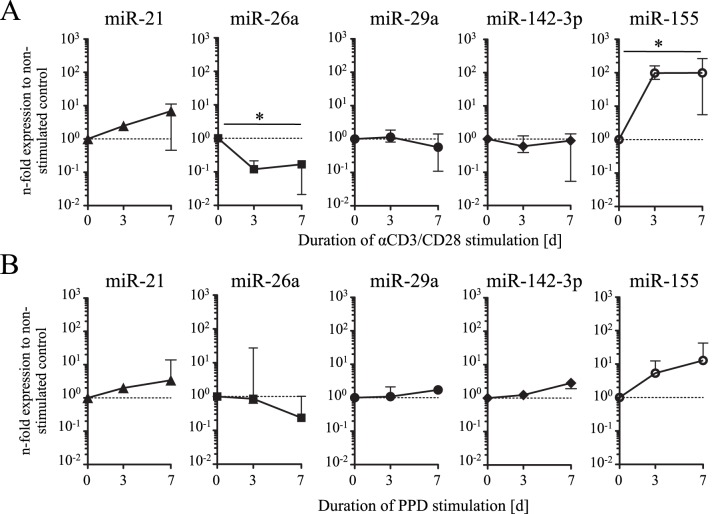
Candidate miRNA expression after activation of naïve T cells and PPD-specific PBMC stimulation. MiRNA expression was analyzed three and seven days after *in vitro* activation of naïve T cells from healthy donors (n = 4) with αCD3/CD28 and for PBMCs of LTBI (n = 3) stimulated with *M. tuberculosis* purified protein derivative (PPD). Expression of the miRNAs was normalized to the housekeeping gene RNU48 and is shown relative to the respective non-stimulated control (dotted line). Median expression and range is shown. Significant differences are indicated as asterisks (* for P<0.05, Kruskal-Wallis test).

In an initial attempt to determine the role of candidate miRNAs in T-cell activation and differentiation, we determined expression of miR-21, 26a, 29a, 142-3p after *in vitro* restimulation. Naïve CD4^+^ T cells were stimulated with crosslinking αCD3/CD28 antibodies *in vitro* and expression of the miRNA candidates was determined. As a control we used miR-155 that is described to be up-regulated upon T-cell receptor-specific stimulation [26]. Following αCD3/CD28 stimulation expression of this control miRNA was increased up to 100-fold already on day three (P = 0.02) (Figure 3A). MiR-21 showed a tendency of increased expression on stimulation (up to 10-fold seven days post stimulation) (P = 0.1) (Figure 3A). MiR-26a expression decreased until day three post stimulation and remained relatively stable until day seven (P = 0.02) (Figure 3). MiR-29a and miR-142-3p showed only moderate expression differences with miR-142-3p being slightly decreased (Figure 3). Notably, similar changes in miR-21 expression were detected after PBMC stimulation from LTBI with *M. tuberculosis*-specific antigen PPD (Figure 3B). A trend of decreased expression was also detected for miR-26a on day seven (Figure 3B). We concluded that miR-21 expression increased upon activation of naïve CD4^+^ T cells *in vitro* whereas miR-26a decreased upon T-cell activation *in vitro*.

### Expression of miR-21, miR-26a, miR-29a and miR-142-3p in Human T_H_1 and T_H_17 Clones

**Figure 4:**
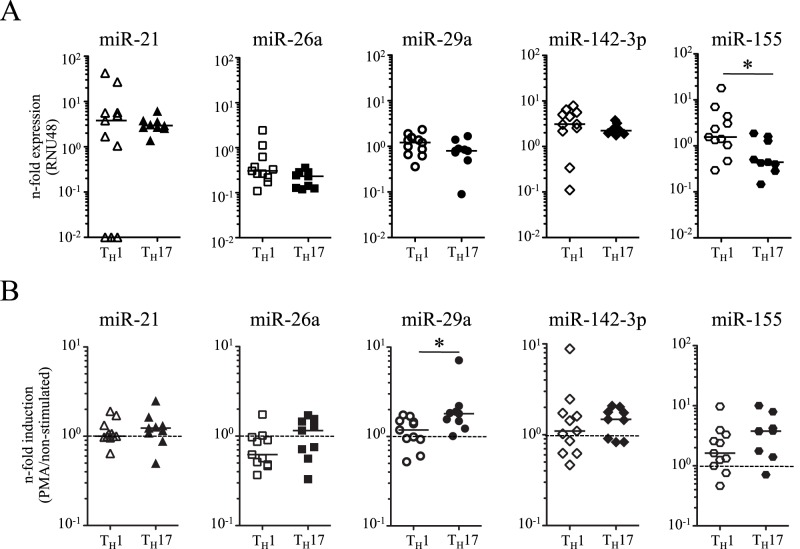
Expression of candidate miRNAs in T_H_1 and T_H_17 clones. Expression of miR-21 (triangles), miR-26a (squares), miR-29a (circles), miR-142-3p (diamonds), and miR-155 (hexagons) was compared between IFNγ-expressing T_H_1 (open symbols, n = 11) and IL-17-expressing T_H_17 clones (black symbols, n = 9) of healthy donors prior to (**A**) and after *in vitro* activation (**B**). Expression of candidate miRNAs was normalized to the housekeeping gene RNU48. Median expression and range is shown. Significant differences are indicated as asterisks (* for P<0.05, Mann-Whitney-U test). (**B**) MiRNA expression after 12 h *in vitro* re-stimulation with PMA/Ionomycin. Relative expression to the respective non-activated control is shown (dotted line).

IFNγ-secreting T_H_1 cells are crucial for protective immunity against *M. tuberculosis* infection [27]–[29]. Polarization of T cells towards functional distinct T_H_-subsets (e.g. T_H_17) may affect susceptibility and protection against tuberculosis. Consequently we analyzed expression of candidate miRNAs in polarized T cell clones. T cell cloning (for details see Methods) resulted in IFNγ-expressing T_H_1 and IL-17-expressing T_H_17 clones. Comparison of miRNA-expression between resting T_H_1 and T_H_17 clones revealed no differences for candidate miRNAs (Figure 4A), whereas miR-155 expression was increased in T_H_1 clones (P = 0.03) (Figure 4A). Notably, activation of T-cell clones with PMA/Ionomycin increased miR-29a expression of T_H_17 clones (P = 0.01) but not of T_H_1 clones (Figure 4B). No differences were detected for miR-21, miR-26a, miR-142-3p and miR-155 upon activation (Figure 4B).

### Similar IFNγ Expression in CD4^+^ T Cells from Children with Tuberculosis and LTBI and No Correlation between IFNγ and miRNA Candidate Expression Level

**Figure 5:**
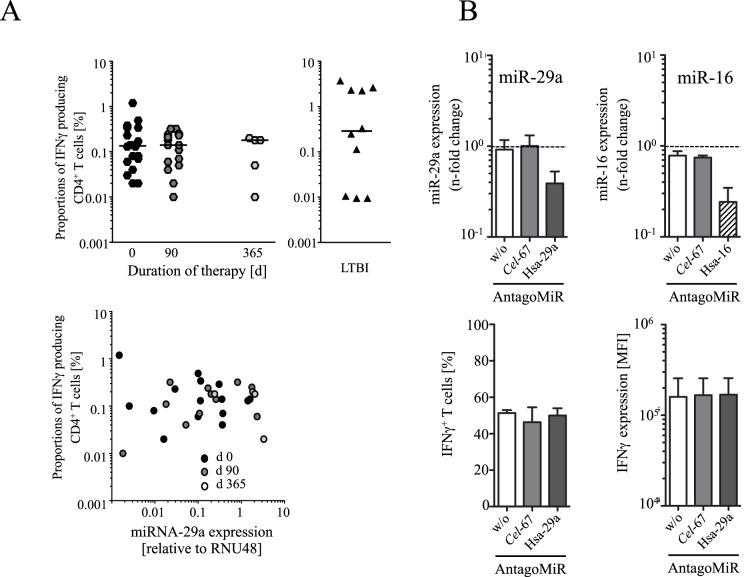
IFNγ and miR-29a expression of infected children and ectopic miR-29a suppression in T cells. (**A**) IFNγ expression after short-term *in vitro* re-stimulation of PBMC from children with tuberculosis (upper left graph) prior to chemotherapy (black symbols), three months under chemotherapy (grey symbols), after recovery (open symbols), as well as children with LTBI (upper right graph) with *M. tuberculosis*-specific PPD is shown. Proportions of IFNγ expressing CD4^+^ T cells (y-axis) for different time points after treatment onset of patients (x-axis) are depicted. Each symbol represents data from an individual patient with tuberculosis (hexagon) or LTBI (filled trigon). *Ex vivo* whole blood miR-29a expression (x-axis) in comparison to the proportions of IFNγ expressing CD4^+^ T cells (x-axis) for individual children with TB under therapy (circles) is shown in the lower graph. (**B**) Modulation of miR-29a expression (upper left graph) and control miR-16 (upper right graph) of CD4^+^ T cells by specific antagomirs (Homo sapiens (Hsa)-29a, Hsa-16, and Caenorhabditis elegans (Cel)-67) and effects on IFNγ expression (lower graphs) are depicted. IFNγ expression of activated CD4^+^ T cells after miR-29a or miR-16 suppression is shown as proportions of IFNγ^+^ cells (lower left graph) or as the IFNγ expression level per cell (indicated by median fluorescence intensities (MFI)) (lower right graph).

IFNγ has recently been identified as a target of miR-29a [21], [22]. Since miR-29a was differentially expressed between children with tuberculosis and LTBI, we compared proportions of IFNγ expressing T cells between these study groups and in children with tuberculosis during treatment and recovery. IFNγ expression was comparable between children with tuberculosis and LTBI after *in vitro* re-stimulation with *M. tuberculosis*-specific PPD (Figure 5A, upper graphs) and also for stimulation with staphylococcal superantigen SEB (data not shown). No differences were detected when comparing IFNγ expression between different time points of children with tuberculosis under chemotherapy and recovery (Figure 5A, upper left graph). Since IFNγ and miRNA expression varied between individuals we also compared expression of IFNγ and miR-29a in individual children with tuberculosis under chemotherapy and after recovery. No dependency between miR-29a and IFNγ expression was detected (R^2^ = 0.03; P = 0.36) (Figure 5A, lower graph).

### Modulation of miR-29a Expression in Primary Human CD4^+^ T Cells

Results of *ex vivo* miR-29a and *in vitro* IFNγ analyses may be confounded by several factors. Therefore, to validate these experiments, we modulated miR-29a expression in primary human T cells. Endogenous miR-29a expression was suppressed using specific antagomir assays (for details see methods). Antagomirs against human miR-16 and *C. elegans* miR-67 have been used as controls in these experiments. Both antagomirs against human miR-16 and miR-29a down-regulated respective targets (miR16, mean 70%; miR-29a, mean 58%) in CD4^+^ T cells, whereas negative control *C. elegans* miR-67 had no effects (Figure 5B, upper graphs). Activation of T cells using PMA/Ionomycin *in vitro* stimulation induced about 50% IFNγ producing T cells (Figure 5B, lower left graph). Notably decreased miR-29a expression did not affect the proportions of IFNγ producing T cells (Figure 5B, lower left graph). Also the cellular protein expression level of IFNγ (determined by single cell median fluorescence analysis) was not influenced by miR-29a suppression (Figure 5B, lower right graph). The same hold true for TNFα producing T cells in this assay (data not shown). We concluded that suppression of miR-29a did not affect IFNγ expression in our assay and therefore other target genes maybe regulated by differential miRNA expression. Especially common targets of the examined miRNAs maybe of interest in this context.

### Common Immunological Targets of miRNAs and PTEN Protein Evaluation Compared to miR-21, 26a, and 29a Expression

**Figure 6:**
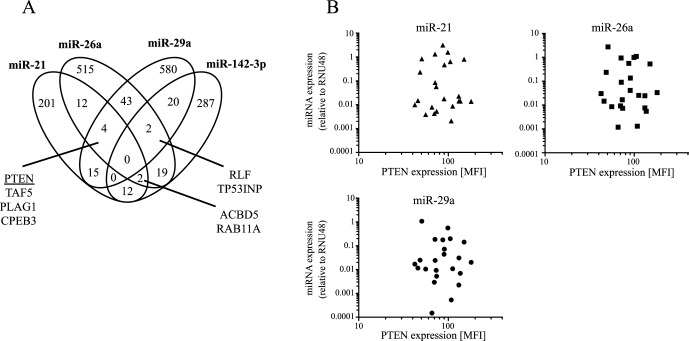
Target genes and overlaps for miR-21, miR-26a, miR-29a and miR-142-3p. (**A**) A Venn diagram indicates the overlap of target genes for miR-21, miR-26a, miR-29a and miR-142-3p. Common targets of at least three candidate miRNAs are listed by name. (**B**) Comparison between expression miR-21 (upper left graph), miR-26a (upper right graph), miR-29a (lower graph) and protein expression of the common target PTEN in lymphocytes from children with tuberculosis (n = 19) and LTBI (n = 3) is shown. We indicate relative miRNA expression to house keeping gene RNU48 by quantitative PCR and compare this to PTEN protein expression determined by flow cytometry using median fluorescence intensity (MFI) analysis. Each symbol represents data from an individual donor.

Predicted miR-21, miR-26a, miR29a, and miR-142-3p targets from the miRGen Internet platform were determined for expression in immunological tissues using Unigene Internet database (for details see Materials and Methods). The predicted candidate miRNA targets expressed in immunological tissues (i.e. 201 genes for miR-21; 287 genes for miR-142-3p; 515 genes for miR-26a; and 580 genes for miR-29a) were then analyzed for overlapping target groups. Figure 6A shows a venn diagram of overlapping miRNA targets. The overall overlap of target genes between the candidate miRNAs was small and not a single gene was predicted target of all four miRNAs. One common target predicted for miR-21, miR-26a, and miR-29a was PTEN, a regulatory protein involved in T cell receptor signaling [30]. Therefore *ex vivo* PTEN protein expression in lymphocytes of children with tuberculosis and contacts was compared. We found no evidence for a correlation of miR-21 (Figure 6B; upper left graph), miR-26a (Figure 6B; upper right graph), or miR-29a (Figure 6B; lower graph) with PTEN protein levels. Ongoing studies aim at validating additional common targets of miRNA candidates to claryfy the role of miR-21, miR-26a, miR-29a, and miR-142-3p expression in tuberculosis.

## Discussion

MiRNAs are involved in the regulation of T-cell immunity and failure may lead to malignancies or autoimmune diseases. The present study provided evidence for a role of miR-21, miR-26a, miR-29a, and miR-142-3p in the immune response against human tuberculosis, a chronic infectious disease. The candidate miRNAs identified in this study were expressed at lower levels in T cells of tuberculosis patients and increased during chemotherapy in individual donors. Analyses of miRNA candidates in whole blood of children with tuberculosis and contacts verified lower miR-26, miR-29a, and miR-142-3p expression in children with tuberculosis. MiR-21 and miR-26a were shown to be regulated during T-cell activation. Initial experiments to modulate miR-29a expression in primary T cells did not suggest a suppressive role of miR-29a on IFNγ regulation.

Host immunity against *M. tuberculosis* is complex and different innate and adaptive immune components are crucial for protection against disease [1]. Because of the unambiguous crucial role of CD4^+^ T cells, we initially focused on this population for identification of candidate miRNAs in the present study. The expression of miR-21, miR-26a, miR-29a, and miR-142-3p was homogeneous within the study groups and already moderate numbers of tuberculosis patients, LTBI, and PPDneg were sufficient to detect significant differences. In addition, the level of differentially expressed miRNAs of tuberculosis patients and controls correlated significantly and therefore, common regulation for miR21, miR-26a, miR-29a, and miR-142 was likely. Previous studies identified key genes for microRNA regulation and a central factor was c-myc [31]. The oncogene c-myc is a transcription factor that regulates several genes and recently it became clear that c-myc also dramatically reprograms miRNA expression [31]. Besides regulating the important miR-17-92 cluster, c-myc has also been shown to suppress miR-26a and miR-29 family members [32], [33]. The related transcription factor n-myc has been identified as a suppressor of miR-21 [34]. Previously we showed that c-myc is up-regulated in T cells from tuberculosis patients prior to therapy [35]. It is tempting to speculate that c-myc is part of the mechanisms leading to concordant down-regulation of miR-26a, miR-29a (and probably miR-21). It remains elusive whether miR-142-3p is also regulated by c-myc. Notably the promotor of c-myc has been identified as a target of nucleoside diphosphate kinase secreted by *M. tuberculosis* [36]. Future studies will address the question if pathognomonic differences in host miRNA expression were caused by direct interaction of *M. tuberculosis* virulence factors with host genes.

MiR-21 and miR-26 showed differential expression after *in vitro* re-stimulation. Here miR-21 was up-regulated and miR-26a was down-regulated after TCR-specific *in vitro* stimulation of naïve T cells and *M. tuberculosis* specific T cells of LTBI. Recently a comprehensive study of miRNA expression in T cells detected decreased expression of miR-26a upon *in vitro* T-cell activation [37]. Our results confirm miR-26a down-regulation after T-cell receptor specific stimulation and likely reflect involvement in T-cell activation and development.

To examine possible roles of the identified candidate miRNAs we performed target gene analysis and focused on targets expressed in immunological tissues (i.e. blood, lymph node, lymph, spleen, thymus). The overlap between target genes was moderate and none of these were predicted for all four miRNAs. Only eight targets were predicted for three candidate miRNAs and classification did not reveal pathways dominantly targeted by miRNA candidates (data not shown). Hence we considered it likely that distinct immunologic processes are involved by differential miRNA expression. A crucial T-cell regulator identified as target of miR-21, miR-26a, and miR-29a is PTEN (phosphatase and tensin homolog deleted on chromosome 10). PTEN is a lipid phosphatase that negatively regulates antigen-activation of T cells by inhibiting the phosphoinositide 3-kinase (PI3K) signaling pathway [38]. Recently Huang et al. found that cell lines deficient for PTEN have an increased susceptibility to *Mycobacterium bovis BCG* infection [39]. Initial experiments have been performed to characterize PTEN protein expression in lymphocytes of children with tuberculosis and LTBI. The fact that we did not detect a correlation between miR-21, 26a, or 29a expression and PTEN protein levels did not exclude a possible role of PTEN regulation since other factors may confound analyses. *In vitro* modulation of miRNA candidate expression is currently performed to verify candidate miRNA targets.

A key factor for susceptibility against tuberculosis is IFNγ and the underlying T_H_1 immune response. It is a matter of discussion if protective T_H_1 immunity against tuberculosis may be impeded by other T helper subtypes (i.e. T_H_2, T_H_17) in a process called immune polarization. Analysis of miRNA candidate expression from *ex vivo* isolated T_H_17 cells was not feasible because of the low frequency of this subpopulation in peripheral blood [40]. Therefore we compared polarized T_H_1 and T_H_17 clones (T_H_2 clones could not be generated) for candidate miRNA expression. Differences were detected for miR-29a that was up-regulated after *in vitro* activation in T_H_17 but not in T_H_1 clones. This is in accordance with previous findings showing an IFNγ suppressive function of miR-29 [22]. In a recent study miR-29a reconstitution alone corrected aberrant IFNγ expression induced in the absence of miRNAs [21]. Furthermore, Ma et al. showed IFNγ itself is a target for miR-29a and that infection of mice with intracellular bacteria led to down-regulation of miR-29a expression in T cells.

In an attempt to characterize the effect of miR-29a expression we compared IFNγ expression in children with tuberculosis and LTBI as well as expression of both (miR-29a and predicted target IFNγ) in lymphocytes of individual donors but detected no dependency between these factors. In addition we determined the influence of miR-29a suppression on IFNγ production of primary human T cells using antagomir technology but did not detect differences of IFNγ expression upon inhibition of miR-29a expression (about 58% reduction). Several reasons may account for these seemingly contrary results. First, approximately 3-fold reduction may not be sufficient for induction of additional IFNγ producing T cells, taking into account that PMA/Ionomycin stimulation activated high proportions of IFNγ expression T cells (about 50% of all T cells). Second, given the dominant T_H_1 response – marked by IFNγ production – induced by *in vitro* stimulation, it is possible that *ex vivo* miR-29a expression levels in T cells were too low to inhibit IFNγ expression. In this case down-regulation would be without consequences but over-expression of miR-29a may lead to IFNγ-inhibition. Ongoing experiments in our laboratory address these questions. Finally, it is tempting to speculate that there are differences in the regulation of IFNγ by miR-29 between animal models and humans. E.g. there may be additional miRNAs that suppress IFNγ in humans and, hence, down-regulation of miR-29a was not sufficient.

We consider the present study an initial step to characterize the role of microRNAs in tuberculosis. Consistent pattern of differentially expressed miR-21, miR-26a, miR-29a, and miR-142-3p suggested a role of these candidates in T-cell immunity during tuberculosis disease and recovery. Further studies will have to be performed to elucidate the processes targeted by candidate miRNAs and to reveal if miRNAs are possible targets for disease intervention strategies in tuberculosis.

## Materials and Methods

### Study Design and Samples

A pilot study was conducted in order to compare miRNA expression among tuberculosis patients and healthy contacts recruited at the University hospital Hamburg-Eppendorf and at the Asklepios Center for Respiratory Medicine and Thoracic Surgery Munich-Gauting, Germany Altogether 16 donors were recruited and peripheral blood (20 ml) was obtained from each donor. Participants were categorized according to the following criteria:

Healthy contacts of tuberculosis patients with a positive T-cell response against purified protein derivative (PPD) of *M. tuberculosis* (Statens Serum Institute) (as described previously [41]) were termed latently *M. tuberculosis* infected (LTBI) (n = 6; median age 32 y; range 29–58 y).

Healthy donors without PPD-specific immunity were termed PPD negative (PPDneg; n = 3).

Diagnosis of active tuberculosis was based on patient history, chest X-ray, tuberculin skin test, QuantiFERON-TB-Gold® test, and mycobacterial culture (TB patients; n = 7; median age 35 y; range 22–52 y).

T-cell clones (TCCs) have been generated using buffy coat cells from anonymous donors. All donors gave written informed consent. The local ethics committee approved this study (Aerztekammer Hamburg, WF-07/09).

In the follow-up prospective cohort study, we recruited 22 children with tuberculosis (median age, 5 y; range 0–16 y), 14 children with LTBI (median age 8 y; range 3–16 y), and 19 non-*M. tuberculosis* infected (PPDneg) children (median age, 3 y; range, 0–11 y) at the Department of Pediatric Pneumology and Immunology, Charité in Berlin, Germany. All children received follow-up examinations three and twelve months after diagnosis. Peripheral blood (max. of 7 ml) was taken at all time points to perform IGRA tests and isolation of peripheral blood mononuclear cells (PBMCs). Of 22 children with tuberculosis initially recruited, 18 were included three months, and of these 13 were included 12 months after onset of therapy. Children were classified according to the following criteria:

Children with LTBI had reported contact to a contagious tuberculosis index case, had positive tuberculin skin test (TST), and Interferon gamma (IFNγ-release assay (IGRA, QuantiFERON®-TB Gold) but showed no clinical symptoms of tuberculosis (including chest X-ray imaging without pathological findings). Children with tuberculosis were diagnosed based primarily on patient history (especially reported contact to index case) and chest X-ray and diagnosis was supported by positive TST or IGRA in all included cases. Children with LTBI and children with tuberculosis were treated according to German guidelines. All legal guardians of included children gave written informed consent. This study was approved by the local ethics committee (EA4/019/09).

### MiRNA Isolation, Reverse Transcription, and Analysis

MiRNAs were isolated from T cells (minimum of 5×10^6^) or frozen blood pellets (of 500 µl heparanized blood) using the *mir*Vana™ miRNA Isolation Kit (Ambion) following manufacturer’s instructions. Reverse transcription was performed with the TaqMan® MicroRNA Reverse Transcription Kit (Applied Biosystems) using two different methods: I) for plate assay analyses using the Megaplex™ RT Primers Human Pool A and B (500 ng RNA each) (Applied Biosystems), II) for single TaqMan® miRNA Assays containing RNA-specific RT primer (100 ng RNA) (Applied Biosystems) following manufacturer’s instructions. For quantitative PCR-based detection of miRNA either a custom-designed 96-well plate (Applied Biosystems) for detection of 29 candidate miRNAs (Table 1) or single TaqMan® miRNA assays (Applied Biosystems) were used. For the 96-well plates reverse transcription method I and for the single assays reverse transcription method II was used. The ‘housekeeping’ control gene RNU48 (official symbol: SNORD48) was applied. The real time PCR was performed in triplicates. Median expression of candidate miRNAs (Ct) relative to the housekeeping gene was calculated (ΔCt). MiRNAs that were detectable in less than 5 tuberculosis patients and 6 LTBI were excluded from further analysis and termed not detectable (nd). For *in vitro* stimulated T cells, ΔCt values of the respective non-stimulated sample were subtracted.

### Enrichment of Total and Naive CD4^+^ T Cells

Peripheral blood mononuclear cells (PBMC) were isolated from 20 ml heparinized blood by density centrifugation (Biocoll, Gibco) and enrichment of CD4^+^ T cells was performed using the magnetic cell sorting system (IMag, Becton Dickinson) following manufacturer’s instructions. The purity of enriched T-cell populations was generally higher than 95% as determined by flow cytometry. Naïve CD4^+^ T cells for *in vitro* stimulation experiments were enriched using ‘untouched’ magnetic cell separation (MACS, Miltenyi) following manufacturer’s instructions and as described before [42].

CD4^+^ T-cell subpopulation enrichment for *ex vivo* miRNA analyses was performed by fluorescence-activated cell sorting (FACS) using the following antibodies: CD4 (APC-H7, clone RPA-T4, BD), CD45RA (FITC, clone HI100, BD), CD3 (PerCP, clone SK7, BD) and CCR7 (PE-Cy7, clone 3D12, BD). Cell sorting was performed using a FACS-ARIA III (BD). A minimum of 2.5×10^5^ sorted cells was acquired and immediately frozen at −20°C for miRNA isolation.

### In vitro Stimulation of T Cells

Naïve T cells (1.2×10^6^) were stimulated with crosslinking αCD3/CD28 Dynabeads (Dynal) (2,5 µl/ml) in 96-well microtiter plates containing 200 µl X-Vivo15 medium (supplemented with 1% Penicillin/Streptomycin). PBMCs of LTBI were stimulated with PPD of M. tuberculosis. Samples were incubated for three or seven days at 37°C and 5% CO_2_. Afterwards cells were immediately frozen at −20°C for miRNA analyses. Short term (overnight) re-stimulation of T cells with *M. tuberculosis* specific PPD for analysis of IFNγ expressing T cells proportions was performed as described previously [41].

### Intracellular PTEN Protein Expression

PBMCs were fixed in para-Formaldehyde (2%) for 10 min at 37°C and were then permeabilized using Phosflow buffer 3 (BD) following manufacturer’s instructions. After single staining with PTEN-specific PE-labeled antibodies (BD) (to avoid confounding effects of multi-color analyses), cells were wash twice and fluorescence intensities were determined using a FACS-Canto flow cytometer (BD). Median fluorescence intensities (MFI) have been shown.

### Generation of T_H_1 and T_H_17 Clones

T_H_1 and T_H_17 clones were generated as described previously [40]. In brief, purified PBMCs (2×10^7^) were stained with monoclonal antibodies (anti-CD4 APC-Cy7, BD and anti-CD161 FITC, Miltenyi Biotec), and CD4^+^ CD161^+^ T cells were sorted into 96-well round bottom plates (1, 2, 3, 5 or 10 cells/well) using a FACS Aria II (BD). Prior to sorting irradiated heterologous antigen presenting cells (1×10^5^ cells/well) and αCD3/CD28 Dynabeads (0,1 µL/well) were added. On day 0, 7, 14, and 21 IL-2 (20 U/ml) was added. After 14 days expanded T-cells clones were counted for each plate. Only T-cells clones from plates with less than 37% positive wells were selected to ensure derivation from a single precursor cell. The cytokine profile of the TCC was determined after PMA/Ionomycin activation over night as described previously [40]. TCC with distinct IFNγ and IL-17 expression were analyzed miRNA candidate levels.

### Modulation of MiRNA Expression Using Antagomirs

Transfection of PBMCs with antagomir-29a and control antagomirs for miR-16, and *C. elegans cel*-miR-67 (all Dharmacon) was performed following manufacturer’s instructions. Briefly, PBMCs (1.5×10^5^ cells/well ) were seeded in 96-well round bottom plates containing X-Vivo15 w/o antibiotics (200 µL/well) and αCD3/CD28 Dynabeads (2 µL/well). After 16 h at 37°C and 5% CO_2_ transfection was done. Antagomirs (5 µM in siRNA buffer (Dharmacon)) were diluted 1∶10 with X-Vivo15 and transfection-solution was prepared mixing DharmaFECT4 (Dharmacon) with X-Vivo15 in a 0.5/10 Ratio. After incubation for 5 min at room temperature (RT) the antagomir-solution was added to the transfection-solution in the ratio of 1∶1 and was incubated for 20 min at RT. X-Vivo15 was added in a ratio of 1∶5. Stimulated cells were centrifuged and the supernatant was discarded. The prepared solution of antagomir, DharmaFECT4, and X-Vivo15 was added (100 µL/well) and the cells were incubated for 48 h at 37°C and 5% CO_2_. Subsequently cytokines (i.e IFNγ, TNFα, IL-2) and miRNA expression was analyzed by flow cytometry and real-time PCR, respectively.

### Identification and Functional Annotation of miRNA Targets

We predicted miRNA targets on the miRGen internet platform (www.diana.pcbi.upenn.edu/cgi-bin/miRGen/v3/Targets.cgi) using PicTar, TargetScanS, miRanda (miRBase), DIANA-microT. Only targets predicted by a minimum of two software algorithms were selected for further analyses. The Unigene EST profile viewer was applied to constrain predicted miRNA candidate targets to those expressed in at least two of five immune tissues (i.e. blood, lymph node, lymph, spleen, thymus). Target annotations and clustering were done using the DAVID internet platform (http://david.abcc.ncifcrf.gov/) Medium classification stringency was chosen and p-values adjusted for multiple comparisons (according to Benjamini-Hochberg) are shown.

### Statistical Analyses

Non-parametric tests have been applied throughout since moderate sample numbers were not sufficient to verify normal distributions. For comparison of miRNA expression between study groups and T-cell clones we applied the Mann-Whitney U-test. The Kruskal-Wallis test has been applied for comparison of miRNA expression in naïve T cells at different time points after *in vitro* stimulation. For correlation analyses of miRNA candidates the Spearman test was used.
